# Supplementary material for: Self-selection biases in psychological studies: Personality and affective disorders are prevalent among participants
Source: PLoS One. 2023 Mar 8;18(3):e0281046. doi: 10.1371/journal.pone.0281046 (PMC9994707; doi:10.1371/journal.pone.0281046)
Supplement: S1 File — (DOCX) [file pone.0281046.s001.docx]

**Supporting Information S1 Methodology File**

The stimulus materials relates to Individually Conducted Face-to-Face Studies with Experimental Manipulation**.**

The research advertisement was disseminated on the same websites as paid survey offers; however, we advertised the study using three different forms of the invitation. Participants could respond either to an advertisement in relation to:

1. a negative critical life event that led to low mood (CLElm Group) and
2. a negative critical life event that did not lead to low mood (CLE Group)

CALL FOR RESEARCH PARTICIPANTS

We invite you to participate in narrative research as part of a research project on the consequences of critical life events funded by the National Science Center.

The invitation is addressed to people aged 22-40 who

- have experienced a negative life event such as: parting with a partner, serious illness, death of a close family member, losing a job, etc
- the event took place less than 2 months before the study
- would like to talk about the event, its meaning and psychological consequences
- **have been experiencing a significantly depressed mood for at least 2 weeks^[[1]](#footnote-1)^**

There will be four meetings: one recruitment and three study meetings during which the respondents will be asked to interview (max. 1 hour) and fill in several questionnaires (approx. 20 minutes).

There will be a remuneration in the form of a shopping voucher in the amount of PLN XX if the respondent is qualified and undergoes the entire research process.

(c) regular (typical) life events and their psychological impact (Normative Group).

CALL FOR RESEARCH PARTICIPANTS

We invite you to participate in narrative research as part of a research project on the consequences of critical life events funded by the National Science Center.

The invitation is addressed to people aged 22-40 who

- **would like to tell about various life events, their meanings and consequences**

There will be two meetings: one recruitment and one study meeting during which the respondents will be asked to interview (max. 1 hour) and fill in several questionnaires (approx. 20 minutes).

There will be a remuneration in the form of a shopping voucher in the amount of PLN XX if the respondent is qualified and undergoes the entire research process.

1. **PLEASE NOTE: This criterion was excluded from the Invitation Form in the CLE Group**  [↑](#footnote-ref-1)
