# Supplementary material for: Self-selection biases in psychological studies: Personality and affective disorders are prevalent among participants
Source: PLoS One. 2023 Mar 8;18(3):e0281046. doi: 10.1371/journal.pone.0281046 (PMC9994707; doi:10.1371/journal.pone.0281046)
Supplement: Attachment — Submitted filename: Resonse to Reviewers.docx [file pone.0281046.s002.docx]

Dear Ankit Jain, M.D.

Academic Editor of PLOS ONE

We would like to thank you for the feedback and helpful suggestions from you and your reviewers. We have complied with all of them. We hope that our manuscript is substantially improved and that you and the reviewers now will find it acceptable for publication. All changes in the manuscript are marked in grey.

Below we present replies to all the comments and suggestions.

------------------------------------------------------------
**Self-selection biases in psychological studies: Personality and affective disorders are prevalent among participants**

1. Please ensure that your manuscript meets PLOS ONE's style requirements, including those for file naming. The PLOS ONE style templates can be found at 
https://journals.plos.org/plosone/s/file?id=wjVg/PLOSOne_formatting_sample_main_body.pdf and 
<https://journals.plos.org/plosone/s/file?id=ba62/PLOSOne_formatting_sample_title_authors_affiliations.pdf>

*Thank you for style templates. Please see that the manuscript meets PLOS ONE's style requirements at this moment.*

2. Please amend your current ethics statement to address the following concerns:
a) Did participants provide their written or verbal informed consent to participate in this study? b) If consent was verbal, please explain i) why written consent was not obtained, ii) how you documented participant consent, and iii) whether the ethics committees/IRB approved this consent procedure.

*In Face-to-Face Studies participants provided their written informed consent to participate. In Online Survey with Volunteers Sample only people who chose to register for the research pool were able to take part (an invitation was sent to them by the pool mailing system). Interested volunteers were informed about the nature and purpose of the study and offered the opportunity to participate. When they chose to participate, they were informed that they could discontinue at any time and their responses would be confidential and not revealed to anyone. They provided informed consent by clicking “Participate” having read the invitation on the research platform.*

3. Thank you for stating the following in the Acknowledgments Section of your manuscript:

"Funding: The first author was supported by grants 2017/01/X/HS6/02022 from the National
Center of Science and BNS 52/20-P. The second author was supported by the grant UMO2017/26/D/HS6/00258 from the National Center of Science and the fourth author was
partially funded by the grant 2019/35/B/HS6/00682 from National Center of Science. The
online study was supported by Ministry of Science and Higher Education in Poland in the
form of subsidy for the maintenance and development of research potential at The Maria
Grzegorzewska University in 2020."

We note that you have provided funding information that is not currently declared in your Funding Statement. However, funding information should not appear in the Acknowledgments section or other areas of your manuscript. We will only publish funding information present in the Funding Statement section of the online submission form. 
Please remove any funding-related text from the manuscript and let us know how you would like to update your Funding Statement. Currently, your Funding Statement reads as follows:

"IK was supported by grants 2017/01/X/HS6/02022 from the National Center of Science and BNS 52/20-P. AZ was supported by the grant UMO-2017/26/D/HS6/00258 from the National Center of Science. PKJ was partially funded by the grant 2019/35/B/HS6/00682 from National Center of Science. The online study was supported by Ministry of Science and Higher Education in Poland in the form of subsidy for the maintenance and development of research potential at The Maria Grzegorzewska University in 2020.The funders had no role in study design, data collection and analysis, decision to publish, or preparation of the manuscript."

Please include your amended statements within your cover letter; we will change the online submission form on your behalf.

*Our amended statement is: "To conduct Face-to-Face Studies IK was supported by grants 2017/01/X/HS6/02022 from the National Center of Science and BNS 52/20-P. PKJ was partially funded by the grant 2019/35/B/HS6/00682 from National Center of Science. The Online Survey with Volunteers Sample was supported by Ministry of Science and Higher Education in Poland in the form of subsidy for the maintenance and development of research potential at The Maria Grzegorzewska University in 2020. The funders had no role in study design, data collection and analysis, decision to publish, or preparation of the manuscript."*

4. Please amend either the abstract on the online submission form (via Edit Submission) or the abstract in the manuscript so that they are identical.

*It is done. Both versions are identical now. Thank you.*

5. Your ethics statement should only appear in the Methods section of your manuscript. If your ethics statement is written in any section besides the Methods, please move it to the Methods section and delete it from any other section. Please ensure that your ethics statement is included in your manuscript, as the ethics statement entered into the online submission form will not be published alongside your manuscript.

*It is done. Thank you.*

6. Please review your reference list to ensure that it is complete and correct. If you have cited papers that have been retracted, please include the rationale for doing so in the manuscript text, or remove these references and replace them with relevant current references. Any changes to the reference list should be mentioned in the rebuttal letter that accompanies your revised manuscript. If you need to cite a retracted article, indicate the article’s retracted status in the References list and also include a citation and full reference for the retraction notice.

*The following positions have been deleted from the References section.*

1. *Beck A, Ward C, Mendelson M, Mock J, Erbaugh J. An inventory for measuring depression. Archives of General Psychiatry. 1961; 4: 561–571.*
2. *Caldiroli A, Capuzzi E, Tringali A, Tagliabue I, Turco M, et al. The psychopathological impact of the SARS-CoV-2 epidemic on subjects suffering from different mental disorders: An observational retrospective study. Psychiatry Research. 2022; 307: 114334.*
3. *Kiejna A, Piotrowski P, Adamowski T, Moskalewicz J, Wciórka J, et al. Rozpowszechnienie wybranych zaburzeń psychicznych w populacji dorosłych Polaków z odniesieniem do płci i struktury wieku–badanie EZOP Polska (Prevalence of selected mental disorders in the population of adult Poles with reference to gender and age structure - EZOP Poland study). Psychiatria Polska. 2015; 49: 15-27.*
4. *Lyon D, Greenberg J. Evidence of codependency in women with an alcoholic parent: Helping out Mr. Wrong. Journal of Personality and Social Psychology. 1991; 61: 435-439.*
5. *Wrosch C, Freund AM. Self-Regulation of Normative and Non-Normative Developmental Challenges. Human Development. 2001; 44: 264-283.*

**Reviewer #1:**

This is an interesting manuscript to review as it highlights that there are significant ways in which research studies are distinct from one another, and these differences have the potential to affect the allure of various studies to various individuals.

The authors try to point out that it is unclear as to the potential personality and affective disorders that may be associated with such choices because people choose the types of psychological studies in which they are willing to participate based on their needs and individual characteristics. This creates an unintentional self-selection bias, but it is important to note that this bias exists regardless.

Most importantly, people who took part in sponsored studies exhibited a greater number of symptoms associated with personality disorders than those who had never before participated for any kind of research. This is a very interesting finding and should prompt researchers to either the alteration of recruiting tactics or much greater caution when generalizing results for this methodological reason.

*Thank you for your feedback.*

**Reviewer #2**

This study presents an interesting concept with a good research question and interesting implications for current research being done in the field of psychiatry. Comorbid personality disorder symptoms can make mood disorders difficult to treat, with patients less likely to respond to medications, more likely to face socioeconomic challenges, likely to suffer from mood symptoms for longer periods of time and hence less likely to comply with their medications. With the most recent research regarding newer antidepressants showing a decline in response to medications and increase in placebo response, this study provides another hypothesis that the patients who are volunteering for these studies may be suffering from additional personality disorder symptoms, which often makes it harder to treat depression. Overall, I found that the study utilized a good method, and the study design appears clear and easy to replicate. The data analysis appears well done, and there does not appear to be any bias or error. The authors can potentially identify actionable points in the conclusion section that institutions and other health care providers can utilize in their own research in order to address challenges posed by these findings.

*Dear Dr Jain Lakshit, thank you for your feedback and sharing your medical perspective.*

*We have added the following paragraph: “Now that we have revealed some serious implications for the conclusions we draw from typical research participants, the next logical question is what can be done about it. We propose three solutions that should not be too onerous. First, we suggest alternative recruitment strategies. For instance, researchers could expressly seek participants who have not participated in research before or include, as a demographic question, how many times in the last month (for instance) a participant took a survey. This might even be something sites like Prolific or Ariadna could provide as part of their meta-data about participants. Second, researchers can engage samples that are more general than the typical psychology student samples including the various Facebook groups for gathering quick data. Third, if the concern is that those who take surveys too often might have psychopathologies, controlling for them with some measure of broadband psychopathologies like the DSM-5 Brief Form PID-5 ((PID-5-BF, American Psychiatric Association, 2013) might be useful. It would allow researchers to ensure that effects found--whether they be correlational or experimental--were not spuriously driven by some psychopathologies. Collectively, these practices would increase the veracity and trustworthiness of findings in much of psychology. While we understand these steps might be annoying and potentially bloating to one's methods a bit, we think the trade-off between in time/effort is worth what will be gained in accuracy”.*

**Reviewer #3**

It was an interesting concept and would be helpful to know more about such kind of biases that might affect volunteers as a whole. It was helpful to have your excellent statistical analysis. The timing especially with comparing non-Covid times to Covid times especially for a volunteer psychology projects seems like it would have added additional confounding factors to the outcome as you have pointed out. It would be interesting to repeat such a study at the same time and with fewer variables being measured.

*Thank you for your feedback. We totally agree that repeating all studies in the same time would be more that highly advisable.*
